# Supplementary material for: DNA-Dependent Binding of Nargenicin to DnaE1 Inhibits Replication in Mycobacterium tuberculosis
Source: ACS Infect Dis. 2022 Feb 10;8(3):612–25. doi: 10.1021/acsinfecdis.1c00643 (PMC8922275; doi:10.1021/acsinfecdis.1c00643)
Supplement: Supplementary file 1 — id1c00643_si_001.pdf [file id1c00643_si_001.pdf]

SUPPORTING INFORMATION (SI)

DNA-dependent binding of nargenicin to DnaE1 inhibits replication in Mycobacterium tuberculosis

Melissa D. Chengalroyen[a§], Mandy K. Mason[a§], Alessandro Borsellini[b], Raffaella Tassoni[b], Garth L. Abrahams[a,c,#], Sasha Lynch[a], Yong-Mo Ahn[c], Jon Ambler[d], Katherine Young[e], Brendan M. Crowley[f], David B. Olsen[e], Digby F. Warner[a], Clifton E. Barry III[c], Helena I.M. Boshoff[c], Meindert H. Lamers[b,*], and Valerie Mizrahi[a,*]

[a]SAMRC/NHLS/UCT Molecular Mycobacteriology Research Unit, DST/NRF Centre of Excellence for Biomedical TB Research, Institute of Infectious Disease and Molecular Medicine and Department of Pathology, Faculty of Health Sciences, University of Cape Town, Anzio Road, Observatory 7925, South Africa.

[b]Cell and Chemical Biology, Leiden University Medical Center, Einthovenweg 20, 2333 ZC Leiden, The Netherlands

[c]Tuberculosis Research Section, Laboratory of Clinical Immunology and Microbiology, National Institute of Allergy and Infectious Disease, National Institutes of Health, 9000 Rockville Pike, Bethesda, MD 20892, USA

[d]Wellcome Centre for Infectious Diseases Research in Africa, University of Cape Town, Anzio Road, Observatory 7925, South Africa.

[e]Infectious Disease, Merck & Co. Inc., West Point, PA 19446, USA

[f]Discovery Chemistry, Merck & Co. Inc., West Point, PA 19446, USA

*Email: m.h.lamers@lumc.nl; valerie.mizrahi@uct.ac.za

Pages: 11

Figures: 6

Tables: 3

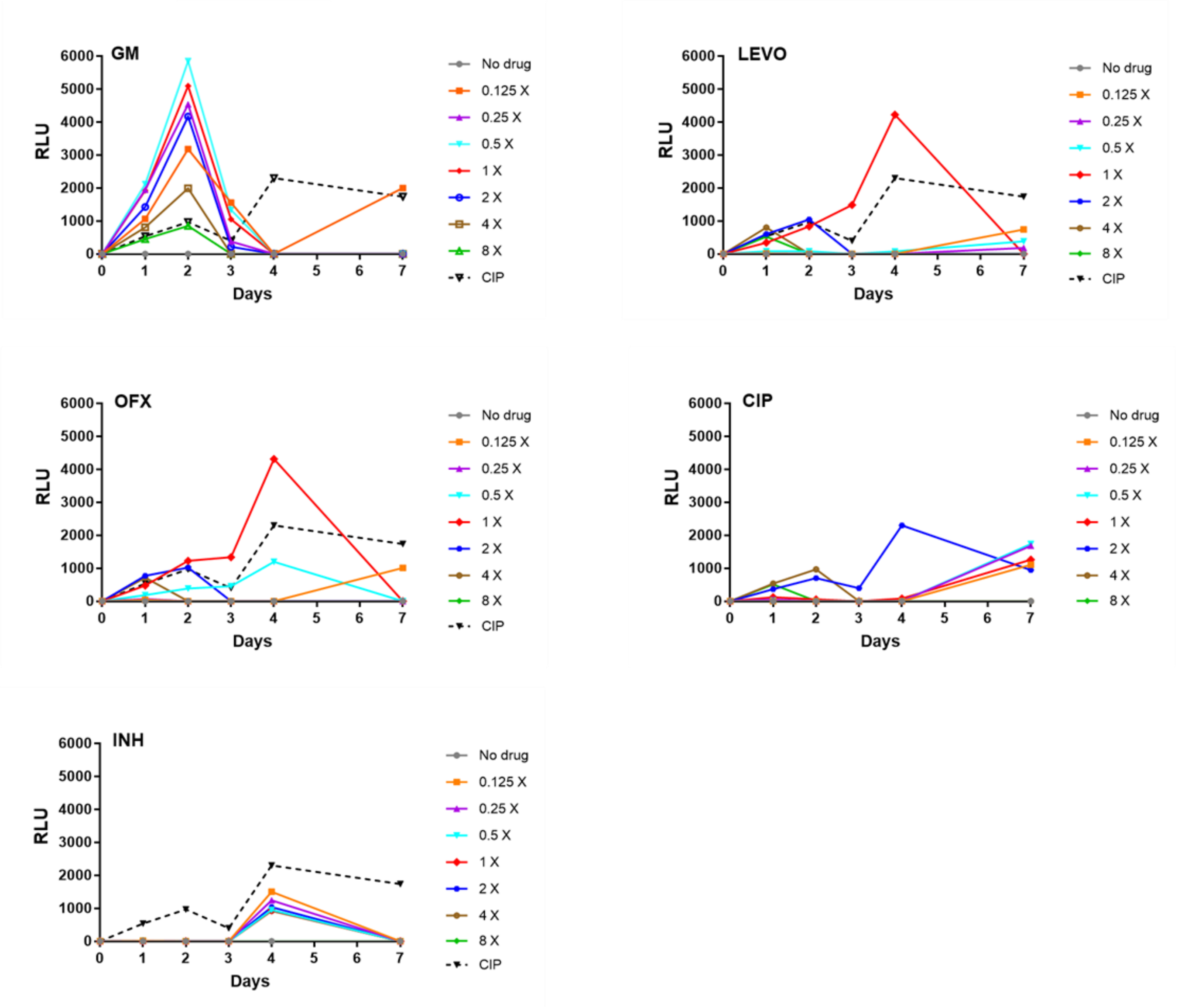

Figure S1. Induction of *recA* bioluminescent reporter, PrecA-LUX [1], in response to various antitubercular agents. Related to Figure 2A. Griselimycin (GM), levofloxacin (LEVO) and ofloxacin (OFX) were tested at concentrations ranging from 0.125× MIC to 8× MIC, and bioluminescence monitored over 7 days. Ciprofloxacin (CIP) was used as the positive drug control at 3.13 µM (2× MIC) and INH as the negative control. Data represent the average of two technical replicates from one representative experiment. Experiments were performed in duplicate.

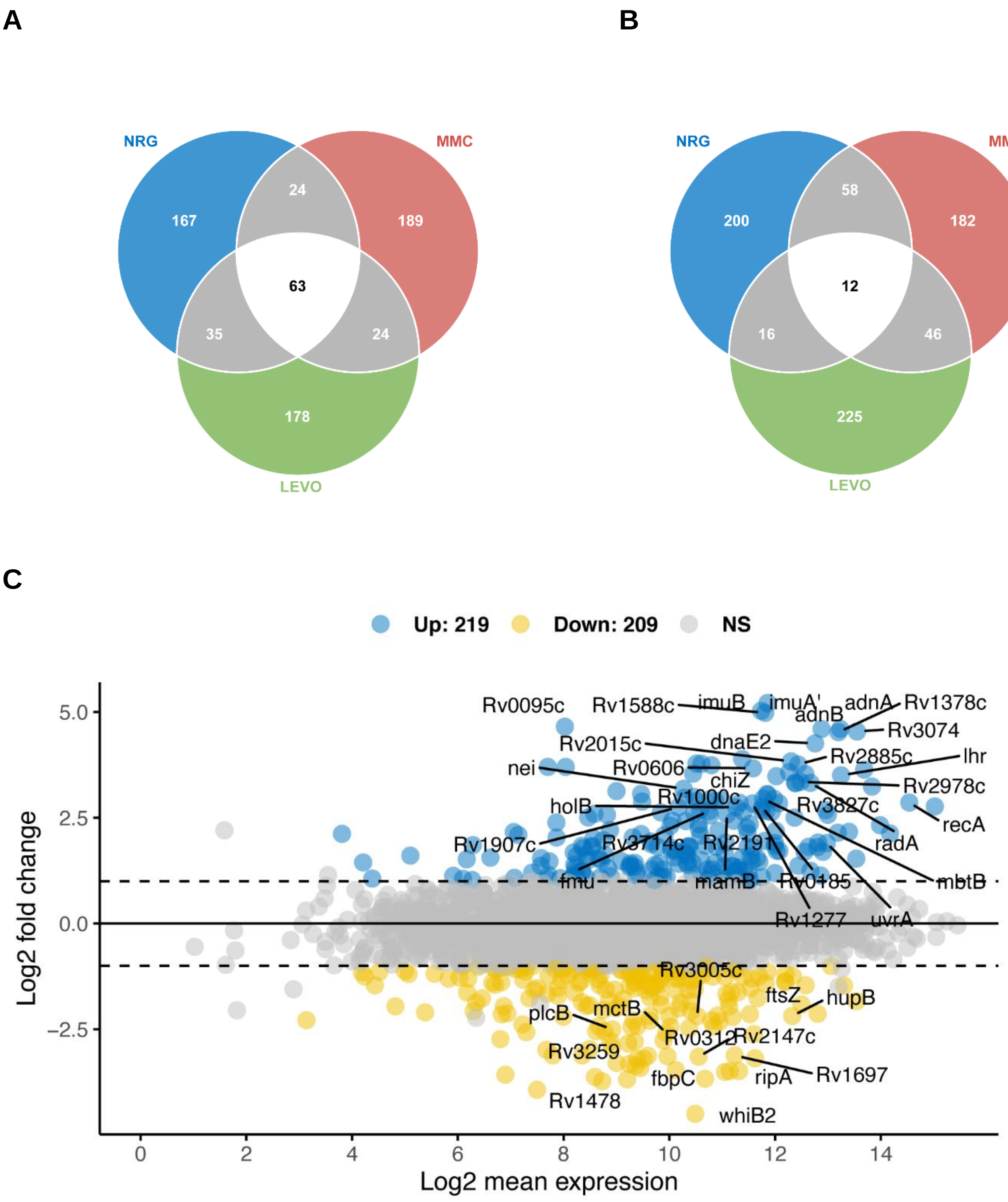

Figure S2. Comparison of the transcriptional response of *Mtb* to nargenicin *versus* other genotoxins. Related to Figure 2B, and Data S1. Venn diagram of the transcriptional response of *Mtb* determined by DNA microarray analysis illustrating the overlap of genes that are either (A) upregulated or (B) downregulated in response to nargenicin (NRG), mitomycin C (MMC) or levofloxacin (LEVO). The genes compared between the three datasets represent the top 300 genes ranked by the average Log2 fold change (FC) in expression data of two biological repeats. (C) Log ratios of RNA-seq data plotted against mean average expression (MA plot) of each gene to visualise the transcriptional response to nargenicin (20× MIC) with genes that show significant upregulation in blue and those that are significantly down regulated in yellow (FDR < 0.05, FC > 2). The genes are listed in the Supplementary Data.

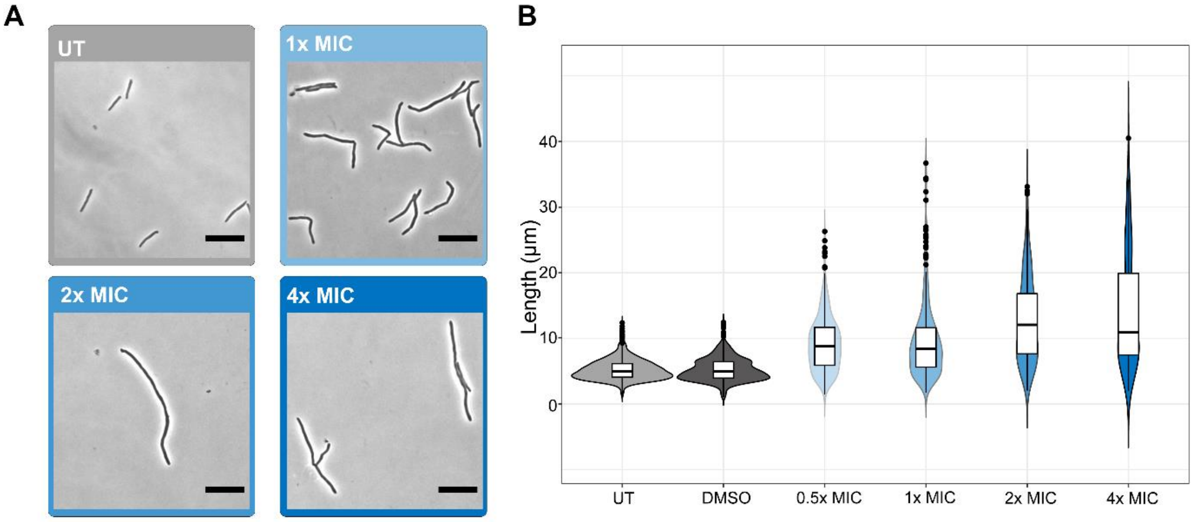

Figure S3. Single-cell length data of from a population of *Msm* bacilli in response to treatment with varying concentrations of nargenicin. Related to Figure 2C. (A) Representative images of untreated (UT) cells and inhibitory nargenicin concentrations, and (B) violin plots showing the distribution of single cell lengths. UT, untreated control; DMSO, carrier-only control. Data for each sample were pooled from two independent experimental repeats. The inset scale bar in panel A represents 10 µm.

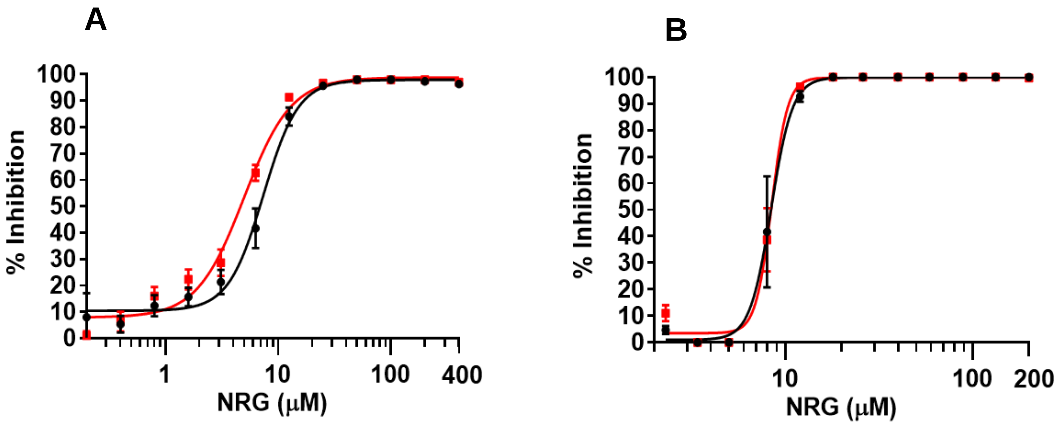

C

Strain	*Msm dnaE1*	*Mtb dnaE1*
mc^{2}155	1.0	-
mc^{2}155 P$_{UV15\text{-}Tet}$-*dnaE1-MYC*::*L5* -ATc	1.2	-
mc^{2}155 P$_{UV15\text{-}Tet}$-*dnaE1-MYC*::*L5* +ATc	8.5	-
H37Rv	-	1.0
H37Rv P$_{UV15\text{-}Tet}$-*dnaE1-MYC*::*L5* -ATc	0.08	1.34
H37Rv P$_{UV15\text{-}Tet}$-*dnaE1-MYC*::*L5* +ATc	2.1	1.3

Figure S4. Effect of *Msm dnaE1* overexpression on susceptibility of mycobacteria to nargenicin. Related to Figure 3C. The recombinant strains (A) mc^{2}155 P$_{UV15\text{-}Tet}$-*dnaE1-MYC*::*L5* and (B) H37Rv P$_{UV15\text{-}Tet}$-*dnaE1-MYC*::*L5* were grown either in the absence (black) or presence (red) of 100 ng/ml ATc and assessed for NRG susceptibility. Bacterial growth inhibition was determined using the fluorescence-based resazurin assay. Means are calculated from three independent biological replicates. (C) Conditional over-expression of *Msm dnaE1* in *Msm* or *Mtb*, assessed by qRT-PCR analysis. Within each strain, quantification of the target *dnaE1* transcript was normalized to the level of endogenous *sigA* transcript. Normalized *dnaE1* transcript levels were divided by the level of expression of the endogenous *dnaE1* transcript to determine relative transcript levels. Comparison of relative transcript levels in the -ATc vs. +ATc samples of the recombinant strains confirmed ATc-dependent induction of *Msm dnaE1* in both *Msm* and *Mtb*. The data represent the results from three independent repeats.

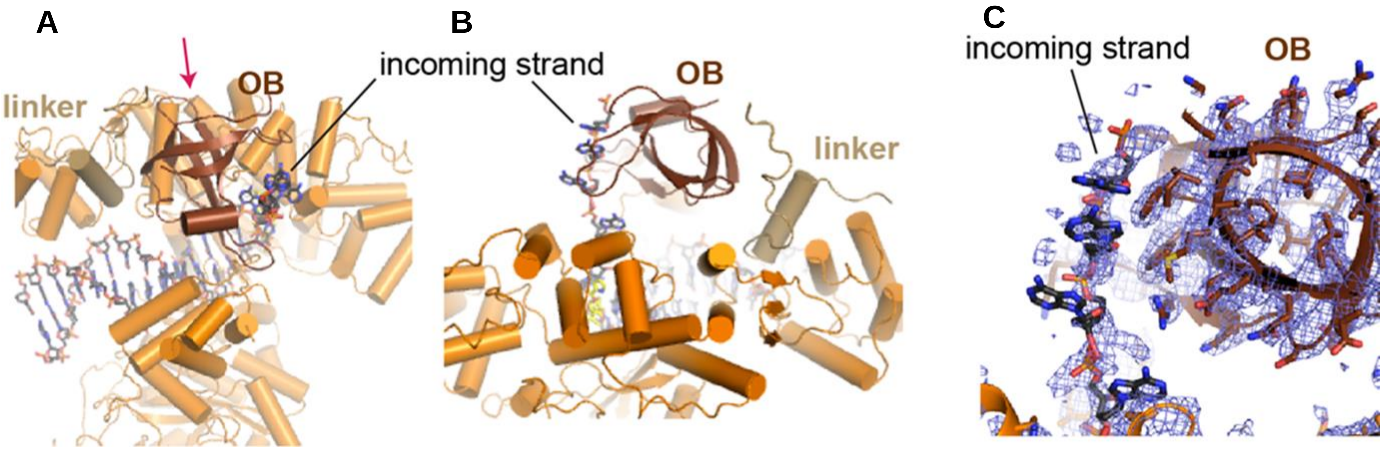

Figure S5. The C-terminal tail of *Mtb* DnaE1 is located on top of the fingers domain. Related to Figure 4. (A) Top view showing the position of the oligonucleotide/oligosaccharide binding (OB) domain (coloured in dark brown) over the polymerase active site and nargenicin (not visible). View is similar to that of main Figure 4B. (B) Close-up of the OB domain and the long linker between the fingers domain and OB domain as viewed along the red arrow in panel a. The connections between the linker and adjacent regions are flexible and not shown. (C) Close-up of the incoming template strand and the adjacent OB domain. The OB domain guides the incoming template strand into the polymerase active site but has little interaction with it. Cryo-EM map shown in blue mesh reveals poorly resolved density around the ssDNA.

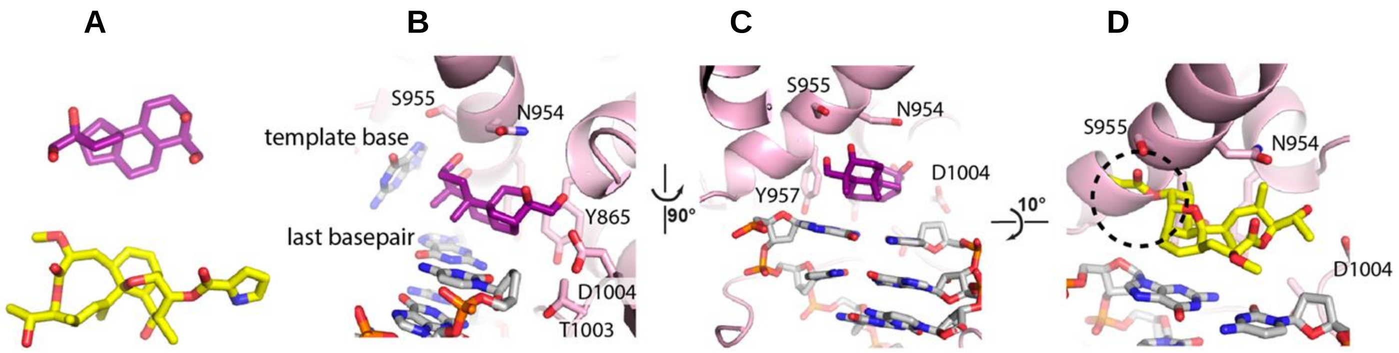

Figure S6. Binding of aphidicolin in human Pol α. Related to Figures 4D, E and 5. (A) Structures of aphidicolin (top, purple) and nargenicin (bottom, yellow). (B) Side view of human Pol α active site showing the position of aphidicolin wedged between the last base pair and the polymerase fingers domain. View similar to that of Figure 3D. (C) Top view of aphidicolin binding. The displaced template base is removed for clarity. View similar to that of Figure 3E. (D) Modelling of nargenicin into the human Pol α active site reveals a major clash between the pyrrole ring of nargenicin and the long helix in the polymerase, marked by a dashed circle. For the structure alignment, the DNA molecules from *Mtb* DnaE1 and human Pol α were superimposed.

Table S1. Antimycobacterial activity of nargenicin. Related to Figure 1B. The activity of nargenicin against different strains of *Mtb* and *Msm* illustrates the effect of media composition on the minimum inhibitory concentration (MIC). Gene names are italicised.

Species	Strain	Media[a]	MIC (μM)
Mtb			
	H37Rv	7H9/OADC/Tw	12.5
	H37RvΔ*dnaE2*	7H9/OADC/Tw	6.3
	H37Rv	7H9/Glu/ADC/T	6.3
	H37Rv	GAST-Fe/Tw	1.6
	H37Rv	GAST/Tw	1.6
	H37Rv	7H9/OADC/Tx	> 100
	H37Rv	7H9/Glu/CAS/Tx	> 100
	CDC1551	7H9/OADC/Tw	31
	HN878	7H9/OADC/Tw	31
	K04b00DS	7H9/OADC/Tw	31
	NIH_KA31b00DS	7H9/OADC/Tw	31
	K14b00DS	7H9/OADC/Tw	63
	K29b00MR	7H9/OADC/Tw	63
	K33b00MR	7H9/OADC/Tw	31
	K37b00XR	7H9/OADC/Tw	31
	K32b00MR	7H9/OADC/Tw	31
Msm			
	mc^2155	7H9/OADC/Tw	50
	mc^2155Δ*recA*	7H9/OADC/Tw	50
	mc^2155Δ*dnaE2*	7H9/OADC/Tw	50

[a]7H9, Middlebrook 7H9 media; GAST/(Fe), glycerol alanine salts (with iron); Glu, glucose; (O)ADC, (oleic acid)-albumin-dextrose-catalase; Tw, Tween, 80; Tx, Tyloxapol.

Table S2. **Genes that are commonly differentially regulated *Mtb* in response to treatment with nargenicin, mitomycin C or levofloxacin, as determined by DNA microarray analyses. Related to Figure S2A and S2B.** Gene lists compared represent the top 300 genes (ranked by the average Log2 fold-change in expression data from two biological repeats. Gene names are italicised.

Genes commonly upregulated by NRG, MMC and LEVO

**Locus**	**Name**	**Description**	**Function**
Rv0054	*ssb*	Single-stranded DNA-binding protein	Essential for replication of the chromosome and involved in DNA recombination and repair
Rv0055	*rpsR1*	30S ribosomal protein S18 1	Implicated in aminoacyl-transfer RNA binding. It appears to be situated at the decoding site of messenger RNA
Rv0056	*rplI*	50S ribosomal protein L9	Binds to the 23S rRNA
Rv0058	*dnaB*	Replicative DNA helicase	Participates in initiation and elongation during chromosome replication; it exhibits DNA-dependent ATPase activity
Rv0181c	Rv0181c	Putative quercetin 2,3-dioxygenase	Function unknown
Rv0336	Rv0336	Conserved 13E12 repeat family protein	Function unknown
Rv0427c	*xthA*	Probable exodeoxyribonuclease III protein	Involved in base excision repair
Rv0515	Rv0515	Conserved 13E12 repeat family protein	Function unknown
Rv0605	Rv0605	Possible resolvase	Prevents the cointegration of foreign DNA before integration into the chromosome
Rv0606	Rv0606	Possible transposase	Thought to be required for the transposition of the insertion element IS1536
Rv0829	Rv0829	Possible transposase	Required for the transposition of the insertion element IS1605'
Rv0922	Rv0922	Possible transposase	Required for the transposition of the insertion element IS1535
Rv1122	*gnd2*	Probable gnd2,6-phosphogluconate dehydrogenase	Involved in hexose monophosphate shunt (pentose phosphate pathway)
Rv1316c	*ogt*	Methylated-DNA--protein-cysteine methyltransferase	Repair of alkylated guanine in DNA
Rv1317c	*alkA*	Probable bifunctional transcriptional activator/DNA repair enzyme	Involved in the adaptive response to alkylation damage in DNA caused by alkylating agents
Rv1377c	Rv1377c	Putative transferase	Function unknown; probably involved in cellular metabolism
Rv1633	*uvrB*	UvrABC system protein B	Involved in nucleotide excision repair
Rv1833c	*dhmA2*	Haloalkane dehalogenase 2	May act on a wide range of 1-haloalkanes, haloalcohols, haloalkenes and some haloaromatic compounds
Rv1957	*secBL*	SecB-like chaperone	Function unknown
Rv2579	*dhaA*	Haloalkane dehalogenase 3	Generates a primary alcohol and halide from 1-haloalkane and H2O
Rv2719c	Rv2719c	Possible conserved membrane protein	Function unknown
Rv2736c	*recX*	Regulatory protein RecX	Binds to RecA inhibiting ATP hydrolysis and the generation of heteroduplex DNA
Rv2737c	*recA*	RecA	Involved in regulation of nucleotide excision repair, in genetic recombination, and in induction of the SOS response
Rv2789c	*fadE21*	Probable acyl-CoA dehydrogenase	Function unknown, but involved in lipid degradation
Rv2790c	*ltp1*	Probable lipid-transfer protein	Possibly catalyses the transfer of a great variety of lipids between membranes
Rv2791c	Rv2791c	Probable IS1602 transposase	Required for the transposition of the insertion element IS1602
Rv2792c	Rv2792c	Possible IS1602 resolvase	Prevents the cointegration of foreign DNA before integration into the chromosome
Rv2977c	*thiL*	Thiamine-monophosphate kinase	Involved in thiamine biosynthesis
Rv2978c	Rv2978c	Probable transposase for IS1538	Required for the transposition of the insertion element IS1538
Rv2979c	Rv2979c	Probable resolvase for IS1538	Prevents the cointegration of foreign DNA before integration into the chromosome
Rv3202c	*adnA*	AdnA subunit of DNA-resecting motor-nuclease	Has both ATPase and helicase activities

Rv3226c	Rv3226c	Putative SOS response-associated peptidase	Function unknown
Rv3296	*lhr*	Probable ATP-dependent helicase, Lhr	Has both ATPase and helicase activities
Rv3394c	*imuB*	Cryptic Y-family DNA polymerase, ImuB	SOS-induced mutagenesis and damage tolerance
Rv3395c	*imuA'*	RecA-like protein, ImuA'	SOS-induced mutagenesis and damage tolerance
Rv3585	*radA*	DNA repair protein RadA	Involved in genetic recombination. May play a role in the repair of endogenous alkylation damage
Rv3586	*disA*	DNA integrity scanning protein DisA	Function unknown
Rv3827c	Rv3827c	Possible transposase within IS1537 element	Required for the transposition of the insertion sequence IS1537
Rv3828c	Rv3828c	Possible resolvase within IS1537 element	Prevents the cointegration of foreign DNA before integration into the chromosome
Rv0057, Rv0059, Rv0060, Rv0184, Rv0185, Rv0959, Rv1277, Rv1702c, Rv1765c, Rv1907c, Rv1945, Rv2015c, Rv2024c, Rv2100, Rv2119, Rv2308, Rv2722, Rv2734, Rv3074, Rv3222c, Rv3466, Rv3642c, Rv3776		Uncharacterized	Unknown

Genes commonly downregulated by NRG, MMC and LEVO

Locus	Name	Description	Function
Rv1698	*mctB*	Copper transporter MctB	Outer membrane channel
Rv2074	Rv2074	F420H(2)-dependent biliverdin reductase	May be involved in biosynthesis of pyridoxine (vitamin B6) and pyridoxal phosphate
Rv2145c	*wag31*	Cell wall synthesis protein Wag31	DivIVA family protein Wag31
Rv2147c	*sepF*	Cell division protein SepF	Part of the divisome complex and is recruited early to the Z-ring
Rv2150c	*ftsZ*	Cell division protein FtsZ	Essential cell division protein that forms a contractile ring structure (Z ring) at the future cell division site
Rv2450c	*rpfE*	Resuscitation-promoting factor RpfE	Factor that stimulates resuscitation of 'dormant' cells
Rv2707	Rv2707	Possible integral membrane protein	Probable conserved transmembrane alanine and leucine rich protein
Rv2986c	*hupB*	DNA-binding protein HU homolog	Histone-like DNA-binding protein which is capable of wrapping DNA to stabilize it
Rv3260c	*whiB2*	Transcriptional regulator WhiB2	Transcriptional regulator of cell division
Rv3330	*dacB1*	Penicillin-binding protein DacB	Involved in peptidoglycan synthesis (at final stages)
Rv1697, Rv3258c		Uncharacterized	Function unknown

Table S3. Primers and other oligonucleotides used in this study. Gene names are italicised.

Primer Name	Sequence (5'-3')	Application
RT-Mtb dnaE1_F	ACCGGACAACTACTTCCTTGA	Forward primer for qPCR analysis of *Mtb dnaE1*
RT-Mtb dnaE1_R	ACACACAACAAAGCCTCATGG	Reverse primer for qPCR analysis of *Mtb dnaE1*
RT-Mtb sigA_F	CGGTGATTTCGTCTGGGATGA	Forward primer for qPCR analysis of *Mtb sigA*
RT-Mtb sigA_R	TGCCGATCTGTTTGAGGTAGG	Reverse primer for qPCR analysis of *Mtb sigA*
RT-Msm dnaE1_F	CGTCGTCTCGTCACTGATCAA	Forward primer for qPCR analysis of *Msm dnaE1*
RT-Msm dnaE1_R	GATGCCCCAACGAATCGAAAG	Reverse primer for qPCR analysis of *Msm dnaE1*
RT-Msm sigA_F	ACCAAGGGCTACAAGTTCTCG	Forward primer for qPCR analysis of *Msm sigA*
RT-Msm sigA_R	CATCTCCTTGGCGAGCTCTTC	Reverse primer for qPCR analysis of *Msm sigA*
Mtb dnaE1sgRNA_3	GAACGCGTAGTCAGCGAACG	sgRNA targeting sequence of *Mtb dnaE1*
Mtb dnaE1sgRNA_6	AGCTGGCCCTCGAAG	sgRNA targeting sequence of *Mtb dnaE1*
Mtb dnaE1 sgRNA_11	GCGGCCGCTTCCACA	sgRNA targeting sequence of *Mtb dnaE1*
Mtb dnaE1 sgRNA_13	GGACCGCCACCTTGGCGTTGA	sgRNA targeting sequence of *Mtb dnaE1*

REFERENCES

(1) Naran, K.; Moosa, A.; Barry, C. E.; Boshoff, H. I. M.; Mizrahi, V.; Warner, D. F. Bioluminescent reporters for rapid mechanism of action assessment in tuberculosis drug discovery. *Antimicrob Agents Chemother* **2016**, *60* (11), 6748-6757. DOI: 10.1128/AAC.01178-16.
